# Immediate and late outcomes of stenting for severe extracranial internal carotid artery stenosis in octogenarian patients

**DOI:** 10.1002/brb3.873

**Published:** 2017-12-20

**Authors:** Jin‐hai Duan, Shu‐wen Xu, Chengbo Dai, Hao Xiao, Jiayi Zhong, Faxin Ma, Jian‐wei Mo, Shuyuan Wang, Xiong Zhang, Zhanyi Lin

**Affiliations:** ^1^ Department of Neurology Guangdong General Hospital Guangdong Academy of Medical Sciences Guangdong Institute of Geriatrics Guangdong Institute of Neurosciences Guangzhou China; ^2^ Department of Neurology Baoan District Central Hospital Shenzhen, Guangdong Province China; ^3^ Guangdong Institute of Geriatrics Department of Geriatric Medicine Guangdong General Hospital Guangdong Academy of Medical Sciences Guangzhou China

**Keywords:** extracranial internal carotid artery, stenosis, stenting, stroke, survival duration RRID: SCR_002865

## Abstract

**Background:**

Multiple studies suggest that internal carotid artery stenting can be performed safely in octogenarians with low periprocedural complication rates. However, great concern still exists as to whether these patients will gain long‐term benefits from this procedure given their advanced age and uncertain life expectancy. We decided to conduct a retrospective study to determine short‐and long‐term clinical outcomes and to analyze survival duration in this population.

**Methods and Results:**

Sixty‐nine consecutive elderly patients with either symptomatic or asymptomatic stenosis ≥70% underwent 86 procedures. Immediate and late outcomes, as well as survival data, were analyzed retrospectively. Mean age was 83.1 ± 2.7 years. Mean survival was 49.3 ± 10.1 months. A complete neurological assessment was obtained at 1 and 2 years in 100% of patients, at 3 years in 90.7% of patients and at 5 years in 84.8% of patients. Two major and one minor ischemic strokes occurred during the periprocedural period. No death, myocardial infarction or intracranial hemorrhage was recorded. The mean follow‐up period was 55.4 ± 24.6 months. Four patients experienced a minimum of 1 year of follow‐up, and the longest is 8 years. Among the patients with the longest follow‐up time, 6 had ischemic strokes, of which 2 were fatal. In total, 17 deaths occurred. Four patients experienced dementia without stroke. Survival at 3 and 5 years was estimated to be 90% and 73%, respectively.

**Conclusion:**

This study demonstrated that stenting in octogenarians was safe and effective during the periprocedural period. Long‐term follow‐up showed a low rate of fatal and nonfatal stroke, and patients survived long enough to benefit from the procedure. However, it was associated with a relatively high rate of long‐term event. Though carotid artery stenting is a minimally invasive procedure, it should still be performed with great caution and only in carefully selected patients. The present study suggested that in this age population, carotid artery stenting might be considered as a revascularization option.

## 1 INTRODUCTION

The incidence of stroke rises significantly in the presence of multiple risk factors (Marini et al., 2004). Advanced age is a leading risk factor for stroke. Its incidence increases from 2% to 7% in patients aged 65 years (Kammersgaard et al., 2004) to 10% in patients aged 80 years. Stroke in octogenarians is the second cause of death and the first cause of permanent disability with a huge impact on healthcare costs and household expenses (Kammersgaard et al., 2004). It is estimated that about 30% to 40% ischemic strokes in octogenarians are secondary to extracranial internal carotid artery stenosis (Gelabert & Moore, 1991). The NASCET trial shows that patients <80 years with high‐grade symptomatic carotid artery stenosis experience up to a 26% chance of ipsilateral stroke at 2‐year follow‐up (Barnett et al., 1991), while those with asymptomatic carotid artery stenosis have a relatively low risk for stroke (Halliday et al., 2004).

Fortunately, for patients <80 years old, carotid endarterectomy (CEA) has been proven to decrease the incidence of stroke in patients with both symptomatic (European Carotid Surgery Trialists’ Collaborative Group, 1991) and asymptomatic (Hobson et al., 1993) carotid artery stenosis disease. In recent years, carotid artery stenting (CAS) has emerged as an alternative procedure for stroke reduction in patients who are considered too high risk to undergo CEA (Gurm et al., 2008). However, CAS in octogenarians is a still a debated subject, given the unsatisfactory results observed in multicenter randomized controlled trials (RCTs) (e.g., EVA 3S,SPACE, ICSS) and the fact that some researchers see increasing age as a predictor of poor outcomes. In very elderly patients (≥80 years old), CAS may be linked to increased periprocedural morbidity and mortality (Hobson et al., 2004; Stanziale et al., 2006). Thus, patients aged over 80 years old were excluded from some clinical trials evaluating CAS (Barnett et al., 1991). The CREST trial reported a 12.1% stroke or death rate at 30 days following CAS in octogenarians. This was not influenced by symptomatic status, lesion severity, and use of distal embolic protection devices (Hobson et al., 2004). Recently, CAS for ICA steno‐occlusion has made great progress in improving the rates of severe disability and death in patients over 80. In effect, reports have demonstrated that CAS can be performed in octogenarians with stroke and death rates comparable to younger patients (Bacharach, Slovut, Ricotta, & Sullivan, 2010; Chiam et al., 2009; Grant et al., 2010; Naylor, 2016; Ouyang, Jiang, Yu, Zhang, & Huang, 2015). A large series of CAS showed a 2.8% stroke and death rate in 30 days and a cumulative 3.3% of major cardiovascular events (stroke, death, or myocardial infarction), which suggested that CAS could be performed safely in patients over 80 (Grant et al., 2010). However, it is uncertain whether these patients would live long enough to stand to gain the most from CAS, a procedure known to have delayed benefits. In order to address this concern, we performed a retrospective study that evaluates the safety and efficacy of long‐term CAS treatment in octogenarians and their postprocedural survival.

## 2 METHODS

### 2.1 Patients

Around 150 CAS were performed each year between January 2008 and December 2015. Sixty‐nine patients aged 80 years or older underwent carotid artery stenting (CAS) in the Department of Neurology of Guangdong General Hospital. The patients were considered for revascularization if diagnosed with symptomatic or asymptomatic ≥70% stenosis. Patients with the following conditions were excluded from our study: dementia, depression, renal dysfunction, anemia, cancer and positive occult test, history of stroke with inability to provide consent or compliance with follow‐up procedures, major surgery before procedure, uncontrolled hypertension, intracranial arteriovenous malformation, coagulopathy, contraindication to use of heparin, or antiplatelet agents. We also excluded patients with aortic arch disease, which would have rendered CAS technically difficult. All patients and their relatives were well informed and signed consent forms.

### 2.2 Procedure

All patients received dual antiplatelet therapy with Clopidogrel (75 mg) and aspirin (100 mg) 3 days before the CAS procedure. Patients who were not on any antiplatelet medication before the procedure were loaded with a dose of 300 mg of Clopidogrel. CAS was performed under conscious sedation with Phenobarbital (100 mg), which was administered 30 min before procedure. Access was obtained at the common femoral artery in all patients under local anesthesia following standard sterile Seldinger technique. After heparinization with an intravenous bolus (100 U/Kg), a 90 cm 8 French guiding catheter was advanced into the distal common carotid artery. Predilation was performed, using an angioplasty balloon only when the stenosis prevented the distal embolic protection device to pass. All cases were performed with distal embolic protection devices through a guiding catheter. The stent delivery system was guided through the stenosis over the guidewire and then deployed. A poststenting balloon dilation was needed when residual stenosis was above 30%. Rate of technical success was 100%.

### 2.3 Cerebral hyperperfusion syndrome

In this study, cerebral hyperperfusion syndrome was defined as follows: (1) Occurrence within 30 days postprocedure; (2) New onset headache, seizure, hemiparesis, and a Glasgow coma scale (GCS) score <15; (3) Cerebral hemorrhage or subarachnoid hemorrhage after CAS or Cerebral Blood flow (CBF) increase of >100% compared with preoperative CT‐perfusion (CTP) values.

### 2.4 Clinical evaluation

Severe ICA stenosis was diagnosed initially by means of Duplex ultrasonography (DUS), CT angiography and MR angiography, and confirmed by conventional angiography (DSA) before carotid artery stenting. The degree of stenosis was calculated, using NASCET criteria (Barnett et al., 1991) in all cases.ECG, echocardiogram and NIHSS evaluation were performed on all patients before CAS. Clinical evaluation was conducted by a neurologist. A major stroke was defined as a new neurological deficit with a change in the National Institutes of Health Stroke Scale (NIHSS) by ≥4 points, which persisted for at least 30 days. Minor stroke was referred to as a new neurological deficit, which showed improvement in the NIHSS by ≤3 points or resolved within 30 days. Prestent and poststent carotid artery stenosis were identified according to NASCET criteria (Barnett et al., 1991). Fatal stroke was referred to as death resulting from either ischemic or hemorrhagic stroke. The periprocedural period was defined as the 30 days after the procedure.

### 2.5 Follow‐up

Outcome and mortality data were collected from inpatient and outpatients records as well as telephone surveys. Patients were available for clinical follow‐up at 1, 3, and 5 years. Patients were questioned regarding new symptoms. If any neurological symptoms were identified at any time after the procedure, the patients would undergo MRI/A or CTA evaluation. All patients received dual antiplatelet therapy for 1 year, followed by treatment with a single antiplatelet and a statin for life. Patients were then followed in our hospital's outpatient clinic in order to address vascular risk factors. The last follow‐up examinations were conducted in December 2016. Patients who underwent carotid stenting at least 1 year before this date were included in the present study.

### 2.6 Ethics approval

This study was approved by our hospital's Medical Ethics Committee.

### 2.7 Data analysis

Data are expressed as mean ± *SD*. The survival rate was estimated using Kaplan–Meier survival analysis. SPSS version 18.0.2 was used for statistical analyses. The authors are fully responsible for the integrity of the data and full access can be granted upon request. All authors have read the entire manuscript and are in agreement with its content.

## 3 RESULTS

### 3.1 Preoperative findings

A total of 69 patients aged 80 years or older were enrolled in this study. Mean age was 83.1 ± 2.7 years (range:80‐93 years old) at the time of the procedure. All had initial severe symptomatic or asymptomatic stenosis and the average degree of stenosis was 78.3%, in accordance with the NASCET criteria. Patients’ demographics, stroke risk factors, manifested symptoms and severity of stenosis are summarized in Table 1. Survival analysis was conducted for all patients, and the mean duration of survival was 55.4 ± 24.6 months postprocedure. Neurological outcomes were assessed at 6 months, 1 year were completed in 100% of the patients, 3 years in 90.7% and 5 years in 84.8%. All patients were available for analysis of neurological outcomes.

**Table 1:** Patient Characteristic

Patients	
Number	69
Male	61
Age(years)	
Mean ± *SD*	83.1 ± 2.7
Range	80–93
Stenosis	
Mean ± *SD*	78.1%
Range	70%–95%
Symptomatic	
Stroke	32
TIA	13
Hypertension	62
Hyperlipidemia	35
Diabetes	19
Ischemic Heart Disease	18

### 3.2 CAS devices

Two different types of embolic protection devices were used in order to perform carotid stenting: AngioGuard XP (Cordis, Miami, FL) (*n* = 49), and FilterWire EZ (Boston Scientific, Natick, MA) (*n* = 20), were used for internal carotid artery stenosis. Two types of stents were placed into the stenotic lesion: Precise (Cordis) (*n* = 41) or Wallstent (Boston Scientific) (*n* = 28).

### 3.3 Periprocedural outcomes

Two patients had a major stroke during the periprocedural period. After treatment, power in the extremities remained 4/5 on the Medical Council Research Scale (MRC). One of the asymptomatic patients developed a minor stroke postprocedure, however, the deficits fully recovered. No death, intracranial hemorrhage or myocardial infarctions occurred (Table 2). Three patients developed a transient bradycardia below 50 beats/min while undergoing dilation and stenting. The heart rate normalized after intravenous injection of 1 mg of atropine. Groin hematoma at the puncture site occurred in two patients and resolved after treatment.

**Table 2:** 30‐day Events

Death	0
Minor Stroke	1
Major Stroke	2
Cerebral hyperperfusion syndrome	0
MI	0

### 3.4 Long‐term outcomes

**Figure 1:**
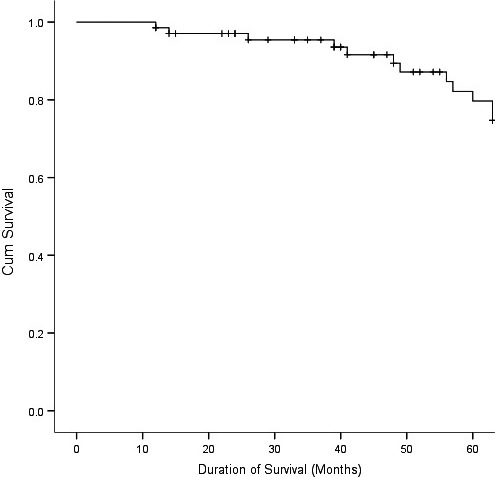
Five‐year survival curve of the entire cohort

**Figure 2:**
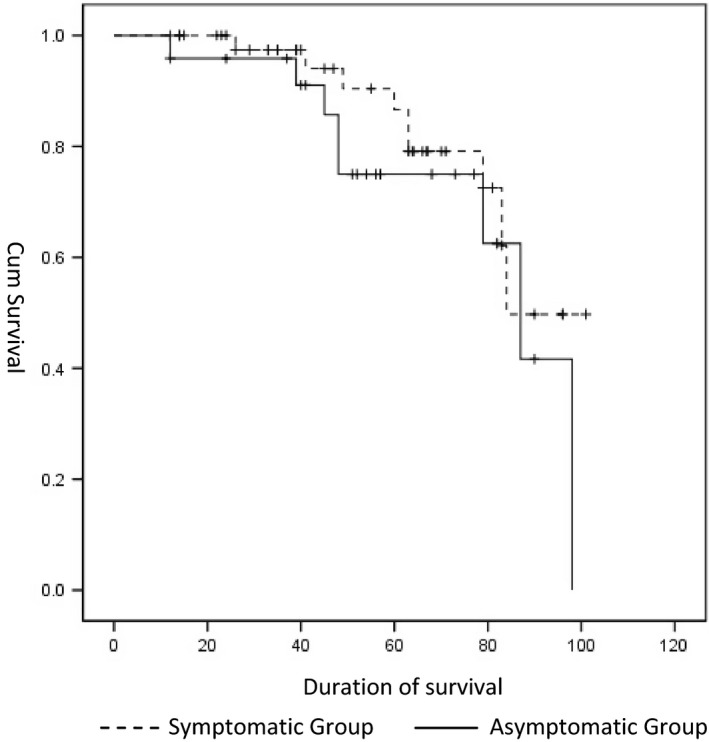
Comparison of survival curve between symptomatic and asymptomatic group

The follow‐up period averaged 55.4 ± 24.6 months (median = 38.6 months). Follow‐up ranged from a minimum of 1 year (*N* = 2) to 8 years. The incidence of postprocedural stroke was 5.6%. No hemorrhagic stroke occurred. Four patients were diagnosed with dementia, however without stroke. Seventeen patients died during the follow‐up period. The cause of death was identified in 11 of the 17 cases. Three deaths were due to cardiac causes; 2 were the result of malignancy; 2 were from fatal strokes; 3 died of pneumonia and COPD; 1 died due to pulmonary embolism (see Table 3).The cause of death for the remaining 6 patients could not be confirmed. Of the entire cohort, 90% were alive at 3 years and 73% survived at least 5 years after the procedure (see Figure 1). Thus, the overall mortality rate for the cohort was 5.4% per year. There was no significant difference in survival rates between symptomatic and asymptomatic patients (*p* > .05) (Figure 2).

**Table 3:** Long‐term Events

Events	Patients(*n* = 69)*n* (%)
Stroke	6 (8.6)
TIA	2 (2.8)
Dementia	4 (5.7)
MI	0
Death	17 (2.6)
Total events	29 (42)

## 4 DISCUSSION

The impact of the patient's age on the success of CAS has been debated for many years. In our study, age was not an important factor in determining the incidence of permanent neurologic deficits during the periprocedural period. Similarly, in other monocentric studies, there is no evidence of a causal link between perioperative neurologic complications and advanced age (Bacharach et al., 2010; Chrysant, Prabhu, Tebow, & Snowden, 2009), although several RCTs showed an increased incidence of complications in octogenarians (Chaturvedi, Matsumura, Gray, Xu, & Verta, 2010; Hobson et al., 2004).

Carotid artery stenting is a safe alternative to CEA, especially in patients with symptomatic carotid stenosis with medical comorbidities who are considered too high risk to undergo CEA. However, there are conflicting results about adverse events associated with carotid stenosis in very elderly patients (Chiam et al., 2009; Hobson et al., 2004; Howard et al., 2016).Early studies showed that adverse events rates in octogenarians undergoing CAS were 25% as compared to 8.2% in younger patients. However, the majority of those complications were minor (Chastain et al., 1999).Roubin et al. found that patients ≥80 years old had a 16% stroke rate in comparison to 5.4% in young patients, and thought that old age (≥80)had a negative influence on periprocedural adverse events Roubin et al. (2001). The lead‐in phase of the CREST trial showed that the periprocedural risk of stroke and death after CAS increased with age and was 12.1% in patients aged over 80, significantly above the rates for patients aged 70–79 (5.3%), 60–69 (1.3%), and less than 60 (1.7%) (Hobson et al., 2004).

Despite these findings, age alone is not an absolute contraindication to Carotid artery stenting. With the aging of the population, it is increasingly common to evaluate octogenarian patients with symptomatic carotid artery stenosis. Elderly patients with symptomatic ICA stenosis always have higher aortic arch complexity, calcification, greater vessel tortuosity and less cerebral reserve in contrast to the younger population. These factors definitely put them at a greater risk during CAS and could lead to poor prognosis. Therefore, improvement in treatment methods is desperately needed in this population. Nonetheless, thanks to improvement in procedural equipment and techniques as well as appropriate case selection, CAS can be performed with lower periprocedural adverse events, falling within the American Heart Association guidelines (Chiam et al., 2008; Grant et al., 2010). Research demonstrates that 85% of the selected elderly patients survived 2 years and ≥75% survived 3 years after stenting. Therefore, for this group, carotid stenting may be considered as a revascularization option (Chiam et al., 2009). But it is still necessary to evaluate the longer‐term outcomes to better understand the role of CAS in this population and to allow them to gain more benefit from this procedure. The present study found that CAS was performed in patients aged over 80 years presenting with severe symptomatic or asymptomatic ICA stenosis, resulting in a low rate of major periprocedural adverse events. The in‐hospital and 30‐day stroke rate was 4.3% and no myocardial infarctions or deaths occurred. These results are similar to a systematic review of outcomes of CEA patients in the general population, which has a 5.1% stroke and death rate in the perioperative period (Bond, Rerkasem, & Rothwell, 2003). Feliziani found that CEA and CAS are safe in elderly patients when cognitive, mood and functional status in the short and long term were taken into consideration for patient selection. CAS is favored because it requires shorter hospital stays, however studies looking at the cost effectiveness of this procedure in older subjects are required.

This study showed that the survival rate at 5 years was 73%, which is similar to the long‐term results observed in the SAPPHIRE trial (Gurm et al., 2008) and in CEA in patients ≥75 years old (Alozairi et al., 2003).

The question remains whether this group of patients will benefit from carotid stenting because they may not live long enough to gain a benefit from the procedure. The NASCET trial initially excluded patients ≥80 years of age (Barnett et al., 1991) and the ACST trial suggested that patients aged 75 years old were unlikely to gain any benefit because mortality was 50% within 5 years (Halliday et al., 2004). Despite these suggestions, CAS is performed increasingly in the aging population without clear survival data. This study shows that 90% of our patients survived up to 3 years and 73% to 5 years. The average mortality rate was similar to the long‐term results reported in the SAPPHIRE trial (Gurm et al., 2008). It suggests that CAS is a reasonable option for most elderly patients because of the high percentage of 3‐year and 5‐year survival rate. However, it should still be performed with greater caution because previous studies have indicated that a longer postprocedural survival is required to gain the benefit (Halliday et al., 2004). Otherwise, total events of long‐term is relatively high to 42% in whole follow‐up period including 4 dementia patients. This result is different from the ACST trial, which concluded that no benefit was achieved in patients ≥75 years of age because of the 50% risk of mortality within 5 years (Halliday et al., 2004). Similarly, ACAS research showed that the ipsilateral and perioperative stroke or death within 3 years was significantly reduced (Executive Committee for the Asymptomatic Carotid Atherosclerosis Study, 1995).

This study provides some information on short‐term and long‐term neurological outcomes of carotid stenting in elderly population. All patients were alive at 1 year and 2 years, with 99% of patients free of new neurological events. The patients who survived to 3 and 5 years after the procedure were free of new vascular events or stroke. At the 5‐year follow‐up, 2 patients died of stroke. These results suggest that after the periprocedural period, the majority of elderly patients are free from cerebrovascular complications. These results are also concordant with a previous large registry data, which demonstrated that, in patients who survive the periprocedural period, freedom from fatal and nonfatal ipsilateral stroke was 99% at 3 years (Roubin et al., 2001). In our study, we explain the lower rates of stroke and death by the fact that we excluded patients with significant medical comorbidities such as abnormal creatine clearance, hemoglobin level and cancer, and by the frequent follow‐ups we conducted to ensure no new conditions arose. Therefore, we had positive results because we performed CAS in relatively healthy elderly patients. A similar population was selected in major CEA studies, which focused on low‐risk patients treated by experienced surgeons and yielded positive results. Thus, if elderly patients are carefully selected, CAS may also yield positive results, with the majority of them likely living long enough to benefit from the procedure.

### 4.1 Limitation

This is a retrospective study performed in a single center. We used rigorous criteria to select our study population. The results may therefore not be generalizable to elderly patients who will not be screened, using the same strict clinical and anatomic criteria. The sample size was small and subgroup analysis could not be performed. Moreover, although we obtained both immediate and long‐term follow‐up, we used different methods to assess patients (review of outpatient records and telephone surveys), which may introduce unforeseen bias. Further multicenter case–controlled studies are needed to confirm the results of the present study. Further studies are also needed to assess the benefit of CAS in the elderly when compared with CEA, as well as to determine long‐term benefits.

## 5 CONCLUSION

This study demonstrated that carotid artery stenting in elderly patients has high efficacy and is safe in the periprocedural period and that patients survive long enough to benefit from the procedure. When selected appropriately, the majority of patients survive to 3 and 5 years after the procedure. In addition, a high percentage was free of stroke, which confirms the efficacy and safety of the procedure. Thus, we argue that CAS is a reasonable revascularization option in the elderly. However, in our study, CAS appeared to be associated with a relatively high risk of long‐term events. Despite this, it remains a good option since it is a minimally invasive procedure that contributes to stroke and disability prevention in the elderly. Adequate patient selection may help improve their expected survival duration. The role of CAS in elderly patients can only be validated through prospective randomized trials comparing stenting with endarterectomy and medical therapy.
